# Scale agreement, ceiling and floor effects, construct validity, and relative efficiency of the PROPr and EQ-5D-3L in low back pain patients

**DOI:** 10.1186/s12955-023-02188-w

**Published:** 2023-09-27

**Authors:** Christoph Paul Klapproth, Felix Fischer, Matthias Rose

**Affiliations:** 1 Center for Patient-Centered Outcomes Research (CPCOR), Department of Psychosomatic Medicine, Center for Internal Medicine and Dermatology, Charité - Universitätsmedizin Berlin, Berlin, Germany; 2 Department of Quantitative Health Sciences, University of Massachusetts Medical School, Worcester, MA USA

**Keywords:** Preference-based measures, Health State Utility, Low back pain, PROPr, EQ-5D-3L, ODI

## Abstract

**Background:**

The PROMIS Preference score (PROPr) is a new health state utility (HSU) score that aims to comprehensively incorporate the biopsychosocial model of health and apply favorable psychometric properties from the descriptive PROMIS system to HSU measurements. However, minimal evidence concerning comparisons to the EQ-5D-3L and the PROPr’s capability to differentiate clinical severity are available. Therefore, the aim of this study was to compare the PROPr to the EQ-5D-3L in terms of scale agreement, ceiling/floor effects, distribution, construct validity, discriminatory power, and relative efficiency (RE) in terms of the Oswestry Disability Index (ODI) for patients with low back pain (LBP).

**Methods:**

We used intra-class correlation coefficients (ICC) and Bland–Altman plots to compare the PROPr and EQ-5D-3L with regared to scale agreement in a cross-sectional routine sample of LBP patients. For distribution, we used the Pearson’s coefficient for skewness and for ceiling/floor effects, a 15%-top/bottom threshold. For convergent validity, we used Pearson’s correlation coefficients. For known-groups validity, we applied a linear regression with interaction terms (predictors sex, age, and ODI level) and an analysis of variance (ANOVA). For discriminatory power, we calculated the effect size (ES) using Cohen’s d and the ratio of the area under the receiver-operating characteristics curves (AUROC-ratio = AUROC_PROPr_/AUROC_EQ-5D-3L_). RE was measured using the ratio of F-values (RE = F_PROPr_/F_EQ-5D-3L_).

**Results:**

Of 218 LBP patients, 50.0% were female and the mean age was 61.8 years. The mean PROPr (0.20, 95%CI: 0.18; 0.22) and EQ-5D-3L scores (0.55, 95%CI: 0.51; 0.58) showed low agreement (d = 0.35, *p* < 0.001; ICC 0.27, 95%CI: -0.09; 0.59). The PROPr’s distribution was positively skewed, whereas the EQ-5D-3L’s was negative. Neither tool showed ceiling/floor effects, but all EQ-5D-3L dimensions did. Pearson correlation was *r* = 0.66 (95%CI: 0.58; 0.73). Differences were invariant to sex and age but not to ODI severity: ES_EQ-5D-3L_ > ES_PROPr_ and RE < 1 in higher ODI severity; ES_EQ-5D-3L_ < ES_PROPr_ and RE > 1 in lower ODI severity. AUROC-ratios did not show significant differences in terms of ODI severity.

**Conclusions:**

All PROPr and EQ-5D-3L biopsychosocial dimensions of health showed impairment in LPB patients. The capability of EQ-5D-3L and PROPr to differentiate ODI levels depends on ODI severity. Joint application of both tools may provide additional information.

## Background

Low back pain (LBP) is one of the most frequent conditions worldwide with a point prevalence of 11.9% [1]. Economically, LBP is the leading health-related cause of productivity loss with a high share of indirect costs (such as absenteeism) [1]. The etiology of LBP is complex and can best be described using the biopsychosocial model of health [1, 2]. If LBP is chronic, which it frequently is, LBP is associated with changes in blood flow and metabolism [2]. Most importantly, behavioral, and emotional factors influence LBP and vice versa, which leads to an increase in the risk of chronic LBP development [2].

It is therefore crucial that measures used for the cost-effectiveness of LBP treatments assess as many biopsychosocial domains of LPB as possible. Economic evaluations of treatments are usually measured in costs per quality-adjusted life years (QALY) gained by an intervention [3, 4]. A QALY is the product of the number of life years and a health state utility (HSU) score (or preference-based measure [PBM]). QALY scores range from 0 (death) to 1 (full health) and represent the value of an individual’s health state. Negative HSU values are considered “worse than dead” [5, 6].

The European Quality of Life 5 Dimensions 3 Levels (EQ-5D-3L), for example, covers five dimensions: mobility, self-care, usual activities, pain/discomfort, and anxiety/depression [7]. Each dimension is measured on a single 3-level Likert-scale item differentiating 3^5^ = 243 health states. These health states were evaluated using preference elicitation techniques that yield a single EQ-5D-3L index value [7, 8]. The EQ-5D-3L shows good psychometric properties in LBP patients, has a low response burden, and is easily applicable [9, 10]. However, on an individual level, its 3-level descriptive items provide coarse measurements, which need to be counterbalanced by large sample sizes. The EQ-5D-3L’s ceiling effects indicate a limited range of measurement. Furthermore, some of its items compose different constructs (e.g., anxiety/depression). Finally, some biopsychosocial dimensions of health (e.g., fatigue), which are potentially relevant to LBP or other conditions, are not part of the EQ-5D-3L [9, 11–16]. The development of its new version, the EQ-5D-5L, improved discriminatory power and reduced ceiling effects [17, 18]. A different approach for covering a comprehensive biopsychosocial LBP model occurred with the introduction of new HSU scores, such as the Patient-Reported Outcome Measurement Information System (PROMIS) Preference Score (PROPr) [19].

The PROPr aims at leveraging the favorable psychometric properties of the descriptive PROMIS system to HSU measurement [12, 19, 20]. PROMIS offers measurement models for health domains (e.g., pain interference) using item response theory (IRT), which allows comparable measurement irrespective of the items used [21, 22]. PROMIS enhances precision and covers a wide range of measurement, mostly showing smaller floor and ceiling effects than comparable measures [11, 23–25]. The PROPr, as a preference-based measure, uses seven PROMIS domains: cognition, depression, fatigue, pain interference, physical function, sleep disturbance, and ability to participate in social roles and activities [26].

Even though the EQ-5D-3L is the most commonly used HSU score, so far, only one comparison to the PROPr has been reported [4, 27–30]. In stroke patients, the EQ-5D-3L and PROPr were strongly correlated [30]. Both scores could differentiate severity levels in terms of modified Rankin Scale [30]. It was suggested that the PROPr is better than the EQ-5D-3L for measuring longitudinal changes at the 1-year follow-up, which, if confirmed, could lead to improved measurements of cost-effectiveness [30]. The authors concluded that the EQ-5D-3L values may be too high for stroke patients in bad health, while PROPr values may be too low for patients in good health [30]. Generally, the PROPr’s face validity as a preference-based measure is disputed as its general population mean was shown to be around only 0.5 on a scale between 0 and 1 [31, 32]. Also, the PROPr with at least 14 but rather 29–33 items poses a higher response burden than the EQ-5D-3L [12, 32–34]. Just recently, the EQ-5D-5L, which is related to the EQ-5D-3L, showed better discriminatory power of physician-diagnosed conditions in a large Hungarian general population sample [31]. However, there is still a lack of analyses of the PROPr’s capability of detecting different severity groups in LBP patients.

The aim of this study therefore is: (1) to assess scale agreement of PROPr and EQ-5D-3L in LBP patients, (2) to compare floor and ceiling effects of both scores, (3) to investigate construct validity in terms of association and different severity groups measured based on the Oswestry Disability Index (ODI), sex, age, and (4) to compare discriminatory power and relative efficiency (RE) in terms of ODI groups.

## Methods

### Sample

We performed a secondary analyses with routine data from a cross-sectional sample of LBP patients before surgery at the multidisciplinary spine center at Charité Universitätsmedizin Berlin. After giving informed consent, patients completed assessment by tablets between April 2019 and November 2020: sociodemographic data, EQ-5D-3L, ODI, and PROMIS-29 profile. All patients presenting with LBP were eligible to participate. Participation was voluntary. No incentives for participation were offered. No explicit exclusion criteria were stipulated. However, illiteracy, vision impairment, and language barriers for non-German-speaking patients were factual barriers for participation. All procedures performed in this study were approved by Charité’s Ethics Committee (EA4/127/16).

### Measures

#### PROMIS Preference Score (PROPr)

The PROPr is a preference-based score based on the PROMIS framework. It covers seven PROMIS domains: cognition, depression, fatigue, pain interference, physical function, sleep disturbance, and ability to participate in social roles and activities [12, 19, 20, 26, 35]. We used the PROMIS-29 v2.0 Profile to measure six of the seven PROPr domains, PROMIS anxiety, and the pain intensity visual analogue scale (VAS) from 0 to 10 [35]. The cognition domain was predicted by a linear regression model from six PROMIS-29 domains [36]. Each domain is measured by four items. Each item is measured on a 5-point Likert-scale and refers to the past seven days, except for physical function, which does not have a specific time frame. These item scores translate into a domain T-score (M = 50 ± standard deviation [SD] = 10) or Theta-score (Z-Scores; M = 0, SD = 1), which is calibrated on the norm of the United States general population [35]. For desirable domains, such as physical function, a higher T-score indicates better health. For undesirable domains, such as pain interference, a higher T-score indicates worse health. Theta scores of all domains were applied to the PROPr multi-attribute utility (MAUT) function to obtain a PROPr between − 0.022 and 1.00, representing the preferences of the US population in 2016 elicited by online standard gamble (SG). Negative values are interpreted as “worse than dead” [12, 19].

#### EQ-5D-3L

The EQ-5D-3L is a preference-based instrument measuring five health dimensions (mobility, self-care, usual activities, pain/discomfort, and anxiety/depression) with one item each on three levels: “no problems” (score: 1); “some/moderate problems” (2); and “extreme problems/unable to/confined to bed” (3). The frame of reference is “Today”. The value assigned to each health state was determined through time trade-off (TTO) in the general population of the United States in 2002 [7]. We used the US value set to avoid systematic differences due to different valuation populations as no German valuation for the PROPr exists yet. A health state of 11111 (namely, each of the five items is answered as ‘1’) has a value of 1.00 (representing perfect health), while the worst health state of 33333 corresponds to a value of -0.103, which is negative and considered “worse than dead” [7]. Last, the EQ VAS item from “The worst health you can imagine” (0) to “The best health you can imagine” (100) measures the patient’s own judgement about his or her health state.

#### Oswestry Disability Index (ODI)

The ODI is a disease-specific index to measure LBP severity. In 10 items with six response options (0–5) for each item, patients rate their disabilities in performing daily routine activities, such as standing and lifting. The sum score is then divided by the maximum sum score of 50 and multiplied by 100, yielding results ranging from 0 to 100%: severity groups are defined as minimal (< 20%), moderate (21%-40%), severe (41%-60%), crippling (61%-80%), and bedridden (> 80%) [37].

### Statistical analysis

First, we investigated scale agreement as based on ICC coefficients using the two-way random effect models and single rater unit. Excellent agreement refers to ICC > 0.75, good agreement to ICC > 0.6, fair agreement to ICC > 0.4, and low agreement to ICC < 0.4 [38]. Our hypothesis was to expect ICC > 0.4 as the PROPr is more comprehensive with regard to the biopsychosocial model of LBP health and has a multiplicative instead of linear-additive utility model. Agreement was further assessed using a Bland–Altman-Plot. Levels of agreement (LoA) of 95% were defined by mean (difference) ± 1.96*SD [39].

Second, we compared ceiling and floor effects both on domain and on HSU score level and the skewness of the distribution of both HSU scores. If more than 15% of the sample scored the maximum or minimum of a scale, this defined significant ceiling or floor effect [40, 41]. To account for skewness of the distribution, we used the Pearson’s coefficient for skewness (γ) in which case if γ < 0, the distribution was negatively skewed and if γ > 0, it was positively skewed [32]. Our hypothesis was to expect no ceiling or floor effects in terms of HSU level but significant ceiling and floor effects in terms of EQ-5D-3L dimensions and none in terms of PROMIS domains. However, we expected γ_PROPr_ > 0 and γ_EQ-5D-3L_ < 0.

Third, we investigated construct validity. For convergent validity in terms of association between the PROPr and the EQ-5D-3L and domains/dimensions, we used the Pearson’s correlation coefficient. Association was defined as strong (*r* > 0.7), moderate (*r* > 0.5), or weak (*r* < 0.5) [42]. Our hypothesis was to expect *r* > 0.5 for the HSU scores and > 0.5 or *r* < -0.5, respectively, for the domains/dimensions [33]. For known-groups validity in terms of sex, age, and ODI severity, we performed a linear regression analyses with interaction terms defined as:

$$HSU=\mathrm{\alpha }+ {\upbeta }_{0}*\mathrm{instrument }+ {\upbeta }_{1}*\mathrm{ODI }+ {\upbeta }_{2}*\mathrm{age }+ {\upbeta }_{3}*\mathrm{sex }+ {\upbeta }_{21}*\mathrm{instrument}*\mathrm{ODI}+ {\upbeta }_{22}*\mathrm{instrument}*\mathrm{age }+ {\upbeta }_{23}*\mathrm{instrument}*\mathrm{sex}$$

Type of instrument (EQ-5D-3L = 0; PROPr = 1) and sex (male = 0; female = 1) were binary variables. Age in years was considered a continuous variable. ODI was measured on an ordinal scale (five severity levels) and dummy-coded. Also, we performed an analysis of variance (ANOVA). As post-hoc test we used the pairwise t-test and adjusted the p-value with the Bonferroni method to further differentiate the differences between ODI severity groups. While we expected significant main effects for instrument and predictor variables, our hypothesis stated that no significant interaction between instrument and age or sex occur, indicating that differences between instruments were not affected by the respective predictors. However, we expected significant interactions between instrument and ODI group.

Fourth, to test discriminatory power between ODI severity groups of both HSU scores and relative efficiency (RE), we used several methods. First, we investigated the effect size (ES) between ODI severity groups of each score using Cohen’s d, which was defined as mean differences divided by pooled standard deviation. ES was considered small (0.2–0.5), medium (0.5–0.8), or large (> 0.8) [43]. Second, we added the ratio of the area under the receiver-operating characteristics curve (AUROC-ratio) as a non-parametric method, using the ODI severity group as outcome and the respective HSU score as exposure for which AUROC-ratio = AUROC_PROPr_/AUROC_EQ-5D-3L_ > 1 indicated higher discriminatory power for the PROPr; AUROC-ratio < 1 for the EQ-5D-3L [44]. As general hypothesis, we expected the PROPr to show a significantly better discriminatory power (*p* < 0.05) between the less severe ODI groups and EQ-5D-3L between the more severe ODI groups in the parametric method comparisons. For the non-parametric method comparisons, we did not expect significant differences. Third, we compared the RE of both instruments to detect differences between ODI groups which was defined as the ratio of F-values: RE = F_PROPr_/F_EQ-5D-3L._ RE > 1 indicates higher efficiency of the PROPr; RE < 1 for the EQ-5D-3L [45, 46]. Since the PROPr domains have more items, we expected that RE would be greater than 1. To calculate the 95% confidence-intervals (CI) for the RE and AUROC-ratio, we used 10,000 bootstrap samples.

## Results

### Sample characteristics

Of 218 patients, 50.0% were female. The mean age was 61.8 ± 17.2 years and ranged from 27 to 92 years. The most frequent ODI severity level was severe. The EQ-5D-3L dimensions of pain/discomfort, mobility, and usual activities were reported to be the most frequently impaired. Accordingly, the PROMIS domains of pain interference, physical function, and ability to participate in social roles and activities were the most impaired. Notably, those domains that are part of the PROPr but not of the EQ-5D-3L (sleep disturbance, fatigue) also showed impairment. The mean PROPr (0.20, 95%-CI: 0.18–0.22) and the mean EQ-5D-3L (0.55, 95%-CI: 0.51–0.58) differed significantly (Table 1).

**Table 1:** Sample characteristics

**Variable**	*n* = 218
Age in years, mean ± SD (range)	61.8 ± 17.2 (27–92)
**Sex, n (%)**	
Female	109 (50.0)
Male	109 (50.0)
**Oswestry Disability Index severity levels n (%)**	
Bedridden	15 (6.88)
Crippling	48 (22.02)
Severe	66 (30.27)
Moderate	59 (27.06)
Minimal	30 (13.76)
**EQ-5D-3L dimension scores, in %**	
Mobility^1^ Level 1/2/3	17.9 / 70.6 / 11.5
Self-care^1^ Level 1/2/3	58.7 / 33.5 / 7.8
Usual activities^1^ Level 1/2/3	22.0 / 59.6 / 18.4
Pain/Discomfort^1^ Level 1/2/3	4.1 / 58.3 / 37.6
Anxiety/Depression^1^ Level 1/2/3	57.8 / 38.5 / 3.7
**EQ VAS**^**6**^**, M (95% CI) [Range]**	51.22 (48.05; 54.40) [0; 100]
**PROMIS-29 domain scores, M (SD)**	
Anxiety^4^	55.94 (10.22)
Cognition^2, 3^	45.37 (3.94)
Depression^4^	54.42 (9.18)
Fatigue^4^	53.55 (10.81)
Pain intensity^5^	6.51 (2.38)
Pain interference^4^	65.32 (7.97)
Physical Function^3^	35.04 (6.81)
Sleep Disturbance^4^	54.23 (8.72)
Ability to Participate in Social Roles and Activities^3^	40.98 (9.35)
**Health States Utilities, M (95%CI) [Range]**	
PROPr	0.20 (0.18; 0.22) [-0.017; 0.869]
EQ-5D-3L	0.55 (0.51; 0.58) [-0.04; 1.00]
Difference (EQ-5D-3L – PROPr)	0.35 (0.32; 0.38)[-0.237; 0.785]

^1^Standard measure to assess general health dimensions developed by European Quality of Life (EuroQoL) group, 1 = no problems, 2 = some problems, 3 = extreme problems

^2^Patient-Reported Outcome Measurement Information System (PROMIS) Cognition was predicted via linear regression from six other PROMIS domains [36]

^3^PROPr PROMIS domains in theta T-score; 50 (population average) ± 10 SD worse/better than population average, higher values indicate better function

^4^PROPr PROMIS domains in theta T-score; 50 = (population average) ± 10 SD worse/better than population average, lower values indicate better function

^5^Pain intensity is measured in visual analogue scale (VAS) with 0 indicating the best and 10 the worst score

^6^EQ VAS EuroQoL visual analogue scale from 0 (worst) to 100 (best), *n* number, *M* Mean, *SD* standard deviation, *CI* confidence interval

### Scale agreement

**Fig. 1:**
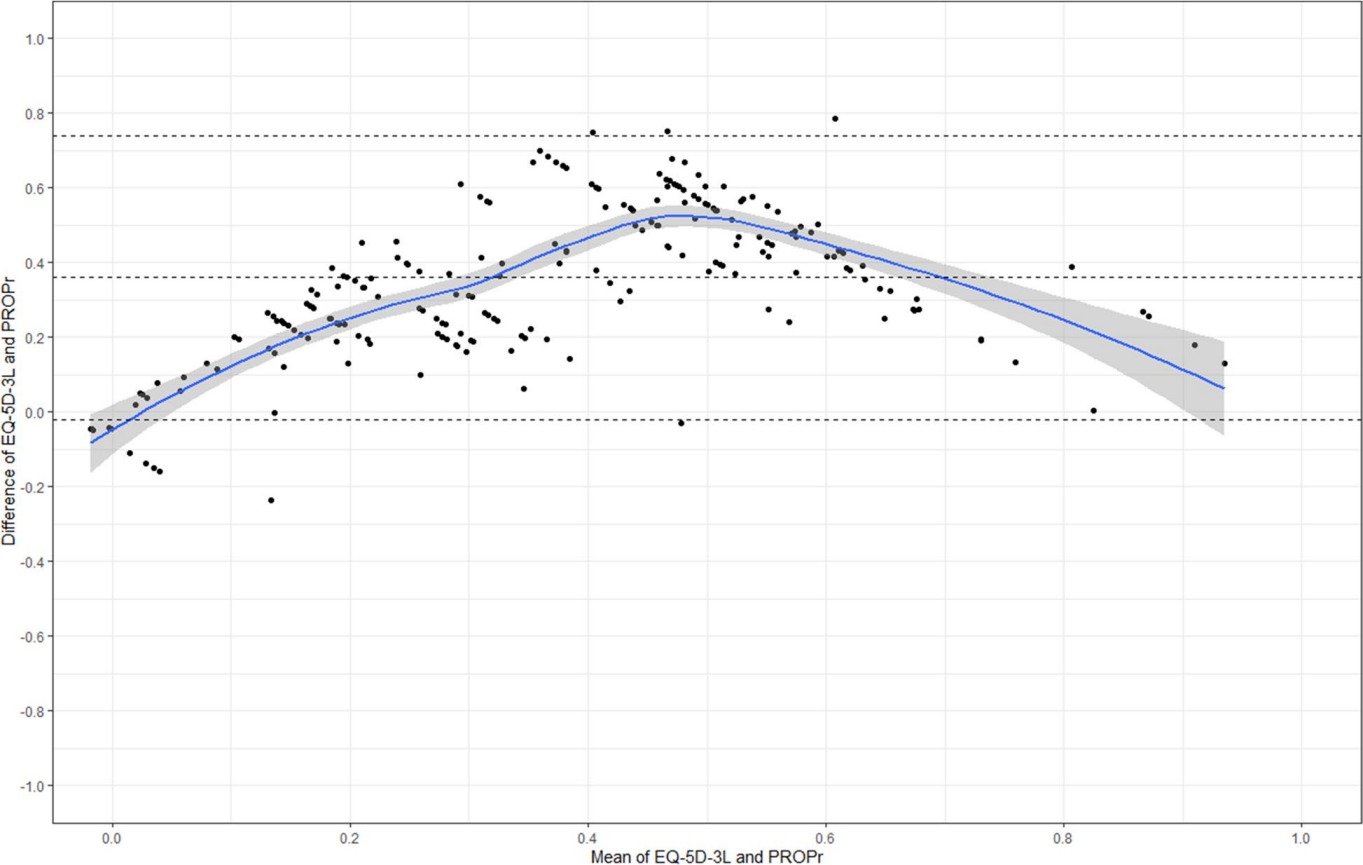
Bland–Altman plot comparing agreement of European Quality of Life 5 Dimensions 3 Levels (EQ-5D-3L) and Patient-Reported Outcome Measurement Information System (PROMIS) Preference Score (PROPr). Upper dashed line: upper 95% limits of agreement (LoA), middle dashed line: mean bias of 0.35, and lower dashed line: lower 95% LoA. The line shows a loess smoother with a 95% confidence interval

The ICC between EQ-5D-3L and PROPr was 0.27 (95%-CI: − 0.09–0.59), which is considered low agreement. The Bland–Altman plot (Fig. 1) demonstrates a systematic difference of d = 0.35 (*p* < 0.001). Agreement was higher for lower and higher values, which is probably an artifact of the bound scales. 95% of HSU differences between PROPr and EQ-5D-3L were between − 0.02 and 0.74, indicating that measurements can differ widely.

### Distribution and ceiling and floor effects

**Fig. 2:**
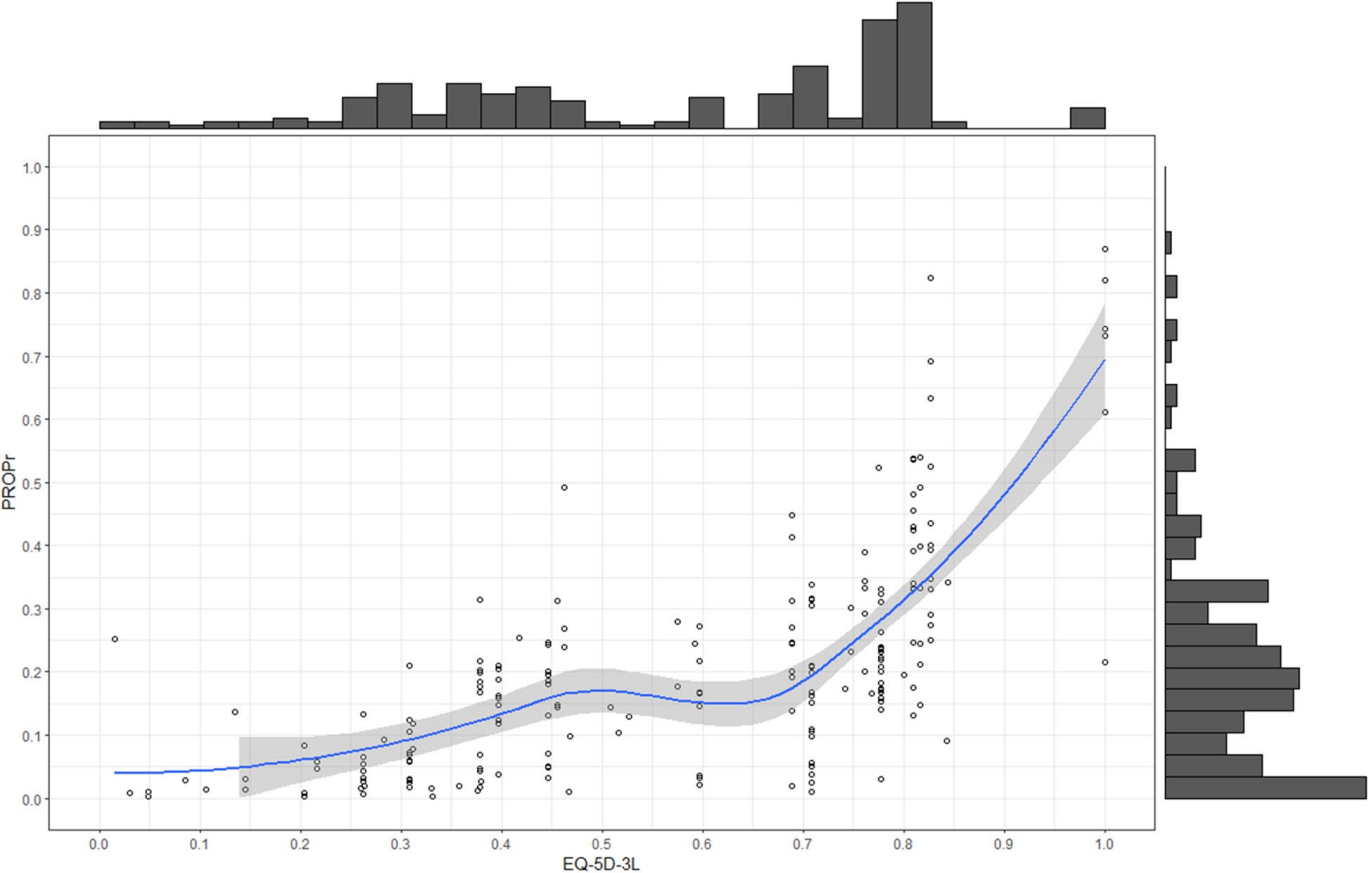
Correlation and distribution of EQ-5D-3L and the PROPr. The line shows a loess smoother with its 95% confidence interval

The PROPr showed a larger positive skew (Pearson’s coefficient for skewness: γ = 1.33) than the EQ-5D-3L’s negative skew (γ =  − 0.55), indicating the former deviates significantly from the normal distribution (Fig. 2).

Table 1 demonstrates that all EQ-5D-3L dimensions, except pain/discomfort, showed ceiling effects. Additionally, pain/discomfort and usual activities showed floor effects. None of the PROMIS scales showed a floor or ceiling effect; in fact, the maxima or minima were never achieved. Neither HSU score showed a ceiling or floor effect.

### Construct validity

For convergent validity, the association measured by Pearson’s correlation coefficient between the EQ-5D-3L and the PROPr was moderate (*r* = 0.66, 95%-CI: 0.58–0.73; Table 2). Likewise, domain/dimension correlations were moderate. Those PROMIS domains showed higher correlation with those EQ-5D-3L dimensions that are conceptually equivalent. PROMIS cognition and EQ-5D-3L anxiety/depression correlated with − 0.59, which is plausible as depression may impair cognition [47].

**Table 2:** Correlation matrix of domains and HSU scores expressed in Pearson’s correlation coefficients r

	EQ-5D-3L	Mobility	Self-Care	Usual Activities	Pain/Discomfort	Anxiety/Depression
PROPr	0.66^***^	-0.58^***^	-0.46^***^	− 0.62^***^	-0.52^***^	− 0.44^***^
Physical Function	0.68^***^	-0.66^***^	-0.56^***^	− 0.59^***^	-0.47^***^	− 0.28^***^
Ability to Participate	0.56^***^	-0.53^***^	-0.45^***^	− 0.63^***^	-0.40^***^	− 0.31^***^
Pain Interference	− 0.64^***^	0.51^***^	0.44^***^	0.57^***^	0.56^***^	0.34^***^
Depression	− 0.42^***^	0.28^***^	0.30^***^	0.32^***^	0.34^***^	0.58^***^
Sleep Disturbance	− 0.22^***^	− 0.10	− 0.01	0.20^**^	0.25^***^	0.33^***^
Fatigue	− 0.35^***^	0.25^***^	0.23^***^	0.36^***^	0.34^***^	0.46^***^
Cognition^1^	0.51^***^	-0.34^***^	-0.30^***^	− 0.44^***^	-0.41^***^	− 0.59^***^

Bold: domains are conceptually equivalent

^1^Cognition was estimated via linear regression

^*^p < 0.1

^**^p < 0.05

^***^p < 0.01

For known-groups validity, a linear regression analyses did not an interaction with age nor with sex but with ODI, suggesting that the observed difference is not influenced by age or sex but with ODI group (Table 3). The ANOVA yielded significant differences in both HSU scores depending on ODI severity. For further differentiation, a pairwise t-test with Bonferroni p-value adjustment found that for the PROPr, all differences except the one between the worst ODI groups “bedridden” and “crippling” were significant (*p* < 0.05). For the EQ-5D-3L, all differences except the one between the best ODI groups “minimal” and “moderate” were significant (*p* < 0.05).

**Table 3:** Coefficients of linear regression analyses with interaction terms predicting EQ-5D-3L

Predictors	HSU (SE)
EQ-5D-3L + age	− 0.0003 (0.001)
EQ-5D-3L + sex	− 0.008 (0.021)
EQ-5D-3L + ODI bedridden	0.141^***^ (0.054)
EQ-5D-3L + ODI crippling	0.227^***^ (0.045)
EQ-5D-3L + ODI severe	0.430^***^ (0.043)
EQ-5D-3L + ODI moderate	0.574^***^ (0.043)
EQ-5D-3L + ODI minimal	0.687^***^ (0.048)
PROPr + age	− 0.001 (0.001)
PROPr + sex	0.008 (0.030)
PROPr + ODI bedridden	− 0.034 (0.077)
PROPr + ODI crippling	− 0.176^***^ (0.063)
PROPr + ODI severe	− 0.312^***^ (0.061)
PROPr + ODI moderate	− 0.343^***^ (0.061)
PROPr + ODI minimal	− 0.240^***^ (0.067)
Observations	434
R^2^	0.731
Adjusted R^2^	0.723
Residual Std. Error	0.150 (df = 420)
F Statistic	87.811^***^ (df = 13; 420)

*HSU* Health State Utility, *SE* Standard Error, *ODI* Oswestry Disability Index

^*^p < 0.1

^**^p < 0.05

^***^p < 0.01

### Discriminatory power and relative efficiency

For PROPr, the ES was large in all comparisons except the one between the two worst ODI groups (Table 4). For the EQ-5D-3L, the ES was large only between the two worst ODI groups. However, ES of both scores presented a large CI, so differences were not statistically significant. Neither was the AUROC-ratio in any of the groups. RE favoured the PROPr statistically significant between “minimal”/ “moderate” and between “moderate”/ “severe”, but the EQ-5D-3L, though not statistically significant, between “severe”/ “crippling” and “crippling”/ “bedridden”. Generally, in all subgroups except ODI group “minimal”, the PROPr had smaller standard deviations (SD) than the EQ-5D-3L.

**Table 4:** Known-groups validity of PROPr and EQ-5D-3L between ODI severity groups in terms of effect size, relative efficiency, and area under the receiver-operating characteristics curve ratio (AUROC-ratio)

ODI Severity level	PROPr	EQ-5D-3L
Scale maximum	1.00	1.00
Minimal Mean (SD)	0.49 (0.19)	0.81 (0.15)
Moderate Mean (SD)	0.26 (0.10)	0.70 (0.15)
Effect Size (95%CI)	1.27 (0.79;1.76)	0.72 (0.26; 1.18)
Relative Efficiency (95%CI)	4.30 (1.68; 19.40)	
AUROC-ratio (95%CI)	1.07 (0.94; 1.24)	
Moderate Mean (SD)	0.26 (0.10)	0.70 (0.15)
Severe Mean (SD)	0.14 (0.09)	0.55 (0.21)
Effect Size (95%CI)	1.07 (0.69; 1.45)	0.74 (0.37; 1.11)
Relative Efficiency (95%CI)	2.52 (1.18; 6.68)	
AUROC-ratio (95%CI)	1.23 (1.00; 1.28)	
Severe Mean (SD)	0.14 (0.09)	0.55 (0.21)
Crippling Mean (SD)	0.07 (0.08)	0.34 (0.18)
Effect Size (95%CI)	0.80 (0.41; 1.19)	0.92 (0.53; 1.32)
Relative Efficiency (95%CI)	0.71 (0.23; 2.02)	
AUROC-ratio (95%CI)	0.94 (0.82; 1.07)	
Crippling Mean (SD)	0.07 (0.08)	0.34 (0.18)
Bedridden Mean (SD)	0.03 (0.04)	0.12 (0.22)
Scale minimum	− 0.022	− 0.103
Effect Size (95%CI)	0.63 (0.03–1.23)	1.04 (0.42–1.64)
Relative Efficiency (95%CI)	0.32 (0.03–2.67)	
AUROC-ratio (95%CI)	0.90 (0.66–1.24)	

ES = 0.2–0.5: small, ES = 0.5–0.8: medium, ES > 0.8: large

*SD* Standard deviation, *CI* Confidence Interval, *ODI* Oswestry Disability Index, *AUROC* area under the receiver-operating characteristics curve

## Discussion

The present study showed that PROPr and EQ-5D-3L measure HSU differently in a sample of LBP patients. Scale agreement is low as neither HSU score shows floor or ceiling effects, but all EQ-5D-3L dimensions and none of the PROPr domains do. The PROPr’s distribution presented a positive skew, while the EQ-5D-3L’s showed a negative skew. Association of both scores was moderate, and differences were invariant to sex and age; however, the EQ-5D-3L could better account for differences in higher ODI severity levels, whereas the PROPr did so in lower ODI severity levels.

The sample size allowed for a relatively high statistical power and generalizability in this patient group for the first part of the analysis. However, for the known-groups validity, when the sample is divided into ODI severity groups, ES, AUROC-ratio, and some RE comparisons were not statistically significant as a result of not having an adequate sample size; thus, our results need to be confirmed in larger samples. Also, unfortunately, our sample lacked enough sociodemographic data and clinical data such as condition and comorbidities. Furthermore, we used the PROMIS-29, so the cognition domain had to be predicted using the linear regression function [36]. A direct measurement is expected to be more precise. Additionally, even though we used US value sets for both scores, the EQ-5D-3L’s was from 2002 and the PROPr’s was from 2016 which may be a source of systematic bias. This cross-sectional psychometric analyses has per se limited validity on how cost-effectiveness analyses differ depending on the HSU scores used in QALY measurements.

The mean difference between EQ-5D-3L and PROPr was 0.41 in a previous study, which is larger than in this present study [30]. Scale agreement comparisons are available only in comparison to the EQ-5D-5L. In terms of ICC, our study’s ICC result was larger (0.48) and mean difference was smaller (0.18) [33]. Three factors cause lower PROPr values compared to the EQ-5D-3L: (1) Generally, the PROPr has more (impaired) domains; (2) The EQ-5D-3L has only three measurement levels, which differs more from PROMIS than the EQ-5D-5L with its five levels; and (3) The PROPr has a multiplicative utility model and relatively large coefficients, which causes interactions between predictor variables. As a result, the PROPr’s HSU scale is in fact narrower than one of the EQ-5D-3L, which has a linear-additive model without interactions. Paradoxically, the PROPr can nevertheless define a higher number of health states which is a product of the levels of the descriptive system and the number health domains [32, 34]. As a consequence, both scores differ to the extent that they cannot be used interchangeably.

Ceiling/floor effects for the EQ-5D-3L dimensions are known and can be explained by its short ordinal scale of only three levels [17, 18, 46]. The EQ-5D-5L therefore has smaller ceiling effects [17, 18]. PROMIS dimensions are measured on a continuous scale and are known to have smaller or no ceiling/floor effects at all even when a different definition is applied [32, 33, 48]. PROMIS domains are explicitly designed to cover a wide range of measurements [35]. This was achieved by using four instead of one item per domain and using items of different severity or difficulty. For example, the PROMIS physical function item “Are you able to run 100 yards?” allows to measure a higher physical function than the correspondent EQ-5D mobility item “Do you have problems walking about?” [19, 20, 30].

The PROPr’s distribution was positively skewed in previous clinical samples as well and approximately normally distributed in general population samples [30, 32, 33]. The EQ-5D-5L and the EQ-5D-3L tend to be rather negatively skewed in any kind of samples [13, 28, 30, 32–34, 40, 41, 44, 46].

Differences in skewness naturally cause a decrease in correlation. The correlation between PROPr and EQ-5D-3L was lower than between the PROPr and the EQ-5D-5L, again because of the lower number of measurement levels [19, 33, 49]. In the one exisiting EQ-5D-3L-comparison, correlations were high (> 0.8) but measured by Spearman’s coefficient [30]. Invariance of the systematic difference between PROPr and other HSU scores with regard to socioeconomic factors, such as age and sex, have been previously reported [32, 33]. Our study is the first to investigate the relationship of these differences to clinical severity in LBP patients. We found that SDs as a measure of precision were smaller for the PROPr than for the EQ-5D-3L scores in the total sample and in all but the “minimal” ODI severity subgroups. This finding is in line with earlier findings in similarly small samples [33]. In larger samples, SD did not differ [32]. The lack of a difference occurred because the PROPr uses four items per domain instead of only one, a process that brings down measurement errors in smaller samples [50, 51]. As the variance has direct impact on F-values, we found that the PROPr’s RE was significantly stronger in the lower ODI group comparisons. The statistical insignificance of comparisons in discriminatory power in terms of ES and AUROC-ratio can be attributed to the small sample size; however, the ES indicated better discriminatory power for the PROPr at lower severity levels and for the EQ-5D-3L at higher severity levels. This finding is in line with recent reports about the differentiation of severity levels in stroke patients [30].

Another study suggested using two rather than just one HSU score in cost-effectiveness analyses to more comprehensively inform decision making [52]. Our results indicate that the PROPr and the EQ-5D-3L could complement one another very well as they have their strengths and limitations at different severity levels, which are at opposite ends of the HSU scale. As PROMIS is increasingly often used as a descriptive measurement of health domains, the necessary data to calculate the PROPr are often available [35]. Further research should be done to investigate this approach.

## Conclusion

All PROPr and EQ-5D-3L dimensions of the biopsychosocial model of health showed impairment in LBP patients. The EQ-5D-3L and the PROPr differ considerably in their measurement and conceptualization of HSU so they cannot be used interchangeably. The PROPr was more efficient and more discriminatory for the lower ODI severity groups, while the EQ-5D-3L was more efficient and more discriminatory for the higher ODI severity groups. Joint application of both tools may provide additional information in cost-effectiveness analyses.

## Data Availability

Upon reasonable request.
